# Effect of Mechanically Treated Recycled Aggregates on the Long Term Mechanical Properties and Durability of Concrete

**DOI:** 10.3390/ma15082871

**Published:** 2022-04-14

**Authors:** Konstantina Oikonomopoulou, Sokrates Ioannou, Pericles Savva, Maria Spanou, Demetris Nicolaides, Michael F. Petrou

**Affiliations:** 1 Department of Civil and Environmental Engineering, University of Cyprus, 1678 Nicosia, Cyprus; oikonomopoulou.konstantina@ucy.ac.cy (K.O.); petrou@ucy.ac.cy (M.F.P.); 2 Department of Civil Engineering, Abu Dhabi Men’s Campus Higher Colleges of Technology, Abu Dhabi 25026, United Arab Emirates; 3 Latomia Pharmakas, 1066 Nicosia, Cyprus; psavva@pharmakas.com; 4 Frederick Research Center, 1303 Nicosia, Cyprus; res.mas@frederick.ac.cy (M.S.); d.nicolaides@frederick.ac.cy (D.N.)

**Keywords:** recycled concrete aggregates, recycled aggregate concrete, adhered mortar, mineral admixes, internal curing, mechanical treatment, mechanical properties, durability

## Abstract

The objective of this research was to study the effect of an optimal mechanical treatment method to reduce the mortar adhered on recycled aggregates (RCA) on the long-term mechanical properties and durability of concretes containing RCA at different replacement levels. It was found that concretes incorporating treated RCA exhibited sharper and more significant increase on 90- and 365-day compressive strengths than any other investigated mixture. The same mixtures also benefitted from a ‘shrinkage-controlling’ effect, where strains and mass losses were reduced by almost 15% and 10%, respectively, compared to the reference concrete. While sulfate resistance and carbonation resistance are predominantly defined by the hydration products available within the cement paste and not to a large extent by the aggregate type and quality, the incorporation of either treated or untreated RCA in concrete did not appear to expose RACs to significant durability threats.

## 1. Introduction

The growth in global urbanization has resulted in an increase in construction and demolition waste (CDW), with concrete being the predominant construction material. In 2018, CDW constituted 35.9% of all total waste in the European Union (EU) [1]. In order to minimize the negative effects of CDW while at the same time preserving the ecosystem and its raw materials, the Waste Framework Directive (WFD) 2008/98/EC and its amendment 2018/851 were endorsed by the European Commission. For all the member states of the European Union, a legislative framework and significant principles for the management of waste were established along with a minimum goal of 70% CDW recycling/recovery/re-use by 2020 [2]. CDW accounts for the highest percentage of total waste in Cyprus, reaching almost 40%, i.e., 10 times higher than the average waste production in the European Union. Moreover, the waste recycling recovery rate in Cyprus is approximately 18% which is low when compared to the majority of EU members [3]. Along with the negative consequences arising from the existence and mishandling of CDW, the continuous depletion of available natural aggregate quantities led to the introduction and utilization of recycled concrete aggregates (RCA) in the construction industry.

RCA consists of natural aggregates (both coarse and fine), old cement mortar which is mainly attached to the aggregates (adhered mortar) and debris (e.g., glass, ceramics, wood, etc.). In comparison with natural aggregates, RCA are defined mostly by a porous microstructure [4], lower density, lack of homogeneity and weaker surface due to the presence of adhered mortar which moderately carbonates the surface [5]. It was understood that the performance of concrete is reduced when high amounts of recycled fine aggregate are present within the mixture [6]. The adhered mortar attached to the surface of RCA influences the new cement paste and the RCA interfacial transition zone (ITZ), creating different and weaker interfacial microstructures, therefore affecting the mechanical and durability properties of the formed material. One of the most prominent characteristics of recycled aggregate concrete (RAC) is the dual ITZ, which is known to provide weaker bonding characteristics in concrete mixtures [5].

In previous research [7,8], the incorporation of RCA in concrete, may or may not lead to poorer mechanical and durability properties. Utilizing RCA in concrete in some studies is shown to slightly decrease the compressive and splitting tensile strengths, although it may still achieve a target strength class of C25/30 [9]. When RCA contents are increasing, concrete is reported to yield lower compressive strengths [10]. Factors such as quality, quantity and treatment method appear to decrease the splitting tensile strength up to approximately 14% mostly due to the presence of old mortar which is partly responsible for the development of weaker ITZs [11]. Similarly, RACs generate lower splitting tensile strength values than those of concretes consisting of natural crushed aggregates [9,12].

Concretes with the same effective water/cement ratio (w/c), cement quantity and 100% recycled coarse aggregates, presented a decrease in compressive strength by 20–25% contrary to normal aggregate concretes, nonetheless, in order to enhance compressing strength values, greater quantities of cement were required [13]. In [14] where 100% RCA were incorporated, compressive strength values were decreased by almost 28% at 28 days of age compared to the reference mixture. However, the authors noted that the reduction at the age of 1 and 5 years was 9% and 6%, respectively. Similarly, splitting tensile strength for 100% RAC was decreased by 10% at 28 days of testing but later increased by 6% and 10% at 1 year and 5 years of testing. From 28 days to 5 years, the increase in compressive and splitting tensile strength was higher related to normal aggregate concretes. The compressive strength of RAC was reduced by 15% and 40% when RCA were incorporated by 50% and 100% respectively according to [15].

Generally, the durability properties of mixtures containing RCA are reported to be impaired in comparison with conventional concretes [9,16]. Higher replacement percentages of RCA in mixtures seem to reduce the electrical resistivity and increase the total charge passing through the examined samples. For mixtures containing 100% RCA, chloride ion penetration had a 20% maximum increase compared to other RAC mixtures with lower replacement percentages of recycled aggregates [16]. Similar behavior was also noticed in fine RACs [11,17].

Comparable results were reported for drying shrinkage experiments when RCA were added [18,19]. Incorporation of inferior quality of RCA along with higher RCA replacement percentages increased the drying shrinkage of concrete [20]. Mixtures with replacement percentages of RCA up to 30%, as opposed to normal aggregates mixtures, presented similar or slightly increased shrinkage results [19,21,22]. A previous study [23] was focused on a modified RAC mixture design in order to achieve improved drying shrinkage results compared to previous research that examined standardized mixture designs with RCA. The addition of 100% RCA in concrete showed an extensive variety in drying shrinkage values, from 10% to 100% higher than their equivalent mixtures containing only normal aggregates.

In other studies [24], RAC mixtures did not exhibit any significant variations in the dynamic modulus when exposed to 5% sodium sulphate solution. Furthermore, compared to natural aggregate (NA) mixtures, RAC mixtures presented decreased values of dynamic modulus [25]. Mixtures containing 100% RCA along with two centrally-embedded reinforcing bars were placed inside a 3.5% Na_2_SO_4_ aqueous solution and examined after 6 months, observing a maximum weight loss of 2.8% [26]. For concrete mixtures containing CEM I [24], the w/c ratio proved to be a determining factor when specimens were immersed in a 5% Na_2_SO_4_ solution. In particular, at a w/c ratio of 0.35 the expansion of samples was decreased, while a relatively high w/c ratio (0.55) had an adverse effect on the expansion was observed.

Overall, when RCA were introduced to concrete mixtures, substantially increased values of carbonation depths were observed in contrast with natural aggregate-based concretes. Specifically, for 25% and 50% RCA replacement, the mean carbonation depth was 1.07 and 1.18 times respectively higher compared to conventional concrete [27]. Furthermore, when mixtures contained 100% RCA, carbonation depths showed a 20% increase compared to other RAC mixtures of lower replacement percentages of RCA in the mixture [16]. When combining CEM I 42.5R and RCA, carbonation depth values were ranging between 1.9–2.1 mm and 2.6–3.9 mm [28]. Moreover, when ground granulated blast furnace slag, fly ash and other supplementary cementitious materials were added into RACs, carbonation depths were comparable to those of reference concrete [29].

To improve the quality of RCA that affects the mechanical and durability properties of RAC mixtures, several treatment methods were developed that mainly focused on the removal of the remnant mortar and/or paste attached to the aggregate’s surface. Removal methods include mechanical treatment [30,31,32], thermal or heating processing [31,33,34,35,36], pre-soaking acid and water techniques [37,38,39,40,41,42], combined methods of heat and mechanical treatment [34,43] etc. Commonly, as expected, treated RAC mixtures mechanical properties such as compressive strength, elastic modulus and drying shrinkage tend to be higher than those containing untreated RCA.

The treatment method adopted for the removal of adhered mortar was studied in [44] and optimized in [45]. Untreated RCA were left rotating inside a concrete drum mixer at 12 rpm along with equals amount of water (weakens the adhered mortar). The impact between the aggregates and the metallic surface of the drum as well as the water presence causes the removal of the old cement paste. Circularity index, mass loss and particle size distribution readings were recorded and examined compared also with natural crushed aggregates where it was concluded that the optimum duration, considering cost and performance evaluation was 3 h. A similar process called autogenous cleaning was also studied in [46] but was conducted for different time intervals.

In light of the previous research work, it is necessary to establish an understanding on the effect of the adhered mortar paste as well as how its quality characteristics could impact the properties of commonly used concrete. Utilizing of the abovementioned methodologies indeed highlights useful information and such methodologies are included in comprehensive reviews. However, there are several concerns embedded with each methodology, such as the energy consumption throughout the treatment procedure, the cost of necessary equipment for running the relevant experiments, the scarcity of facilities for conducting essential rigorous advanced techniques, as well as risks of jeopardizing the existing aggregate properties used in the aforementioned research. It is therefore imperative, when utilizing a particular methodology towards enhancing RCA, to determine an optimum balance between the cost embedded with the usage of relevant equipment (i.e., relevant instrumentation, cost of energy associated); the environmental impact associated with the conducting of experimental procedure (i.e., embodied CO_2_ carbon footprint, quantitative energy consumption), and the overall performance yielded from the experimental data.

The objective of this research is a continuation of the work conducted by Savva et al. [45] to conduct an optimal treatment on RCA and incorporate those in RAC while examining whether this would enhance the long-term mechanical and durability properties of conventional and high strength concrete. The partial removal of the adhered mortar was achieved using a mechanical treatment method involving a truck drum mixer as reported in [45] and the effect of such reduction on the mechanical and durability properties of concretes incorporating RCA at variable replacement percentages is investigated. Indeed, mechanical treatment provides advantages towards reducing the adhered mortar from RCA, such as the elimination of the existing weakly-binding mortar and the avoidance of the formation of poorly sustained ITZs within the microstructure. On the other hand, it also causes a negative effect as well, i.e., increasing the aggregate circularity index, thus leading to a decrease of aggregate interlocking in concrete—effectively reducing the bonding, between aggregate and mortar, and adversely affecting concrete’s mechanical properties. The above concerns need to be taken into consideration, along with the environmental impact and the associated cost for yielding equivalent performance to that of commonly used concrete. The specific evaluation is currently being studied in detail using a techno-economic and life cycle assessment. The results will be discussed and published at a later stage. The results of our previous work [45] suggest that both cost-effectiveness and practicality of the examined method were beneficial towards producing a cost-effective RAC to yield equivalent performance to that of conventional concrete. In this light, the current investigation aims to extend upon this work by studying the effect of an optimal duration of mechanical treatment where RCA were brought to the most optimal geometrical state on the long-term mechanical and durability properties of RAC.

Section 2 refers to the materials used in the experimental work for developing appropriate RAC, as well as the formulations developed at incremental additions of recycled aggregates and to the series of experimental tests conducted on the particular mixtures. Section 3 presents the results of the mechanical and durability testing programme, followed by concluding remarks on the effect of mechanically treated RCA on the long-term performance of RAC.

## 2. Materials and Methods

**Figure 1:**
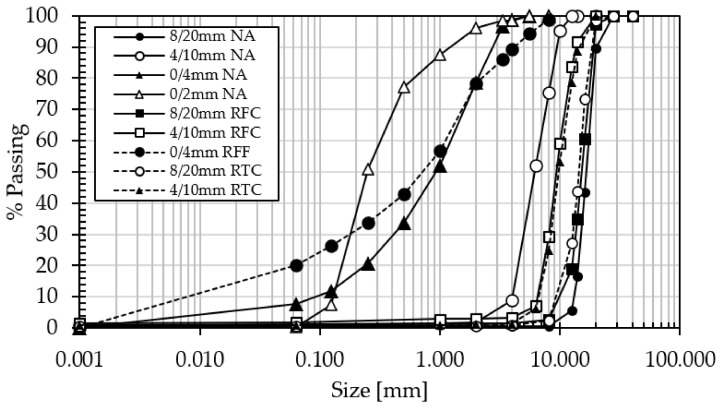
Particle size distribution of the aggregates used in the experimental program [52].

Diabase natural aggregate and recycled aggregates from unknown sources as obtained from demolished structures in various parts of the country, were obtained from the Pharmakas quarry in Cyprus, with sizes of 0/4, 4/10 and 8/20 mm. The properties of the materials were determined in accordance with the relevant EN Standards [47,48,49,50,51] as shown in Table 1. The particle size distribution of the aggregates is shown in Figure 1. A petrographic analysis of the natural diabase aggregates revealed that the material stems from finely to medium crystalline igneous rock of mainly tectosilicate, amphibole, chlorite, zeolite basalt or dolerite structures.

### 2.1. Mechanical Treatment of Aggregates

The treatment method was based on the mechanical partial removal of the adhered mortar from the surface of the RCA, resembling a prolonged Los Angeles test. A 10 m^3^ capacity concrete drum mixer was used for rotation at 12 rpm along with water for the aggregates to collide with each other and thus altering the geometrical and morphological characteristics by partially eliminating the weakly bound adhered mortar. The procedure involved the determination of the mass loss and circularity of aggregates using imaging analysis and examining the effect of the duration of such treatment on the changes in the values of the circularity index. The procedure can be found in detail in our previous work [45].

### 2.2. Concrete Properties Investigated

The study includes evaluating the mechanical properties and durability of the concretes incorporating RCA at strategic replacement contents. Table 2 provides the test type, relevant standards used, and age of testing in the experimental. For determining the mechanical properties of the mixtures, compressive strength according to EN 12390-3 [53] and splitting tensile strength according to EN 12390-6 [54] were conducted using a 5000 kN capacity CONTROLS Advantest-9 hydraulic compression machine on three samples for each test. Durability tests focused mainly on the ion transport properties of concrete. Capillary water absorption (sorptivity) in accordance with RILEM TC-116 [7] was conducted, where 5 mm slices were cut from 100 mm cubic samples (three samples for each formulation tested) in order to expose the aggregate cross-section. The samples were initially placed at 110 ± 5 °C until constant mass, then cooled off to reach room temperature and sealed with an elastomer-based impermeable coating in all surfaces apart from the exposed one. The exposed surface was then immersed at a depth of approximately 3 mm of 2-propanol. The change in mass of the samples was determined at specific intervals of square root of time and finally the sorptivity coefficient was determined based on the function slope using the equation below:

Q/(Aρ) = kt^0.5^ (1)

where Q = the amount of 2-propanol absorbed (g), Ρ = the density of propanol (g/mm^3^), A = the cross-section area exposed to propanol (mm^2^), t = the time (min) and k = the sorptivity coefficient (mm/s^0.5^).

The open porosity [7] of concrete mixtures was determined by initially oven-drying the samples at 65 ± 5 °C until constant mass (m_d_), following by placing them in a vacuum desiccator subjected to a pressure of 2.0 ± 0.7 kPa for a duration of 2 ± 0.2 h. The procedure included filling the desiccator with demineralized water following which the atmospheric pressure was restored. The samples (three samples for each tested formulation) were maintained within the desiccator for 24 h and both immersed (m_h_) and saturated surface dry (m_s_) masses were measured. The open porosity (%) was then determined by calculating the ratio of the open pore volume and the apparent volume of the samples, based on the equation below:

ρ_ο_ = [(m_s_ − m_d_)/(m_s_ − m_h_)] × 100 (2)

Chloride resistance of 28, 56, 90 and 365-day cylindrical concrete disc specimens of 100 mm diameter and 50 mm height (cut from 100Φ200 mm cylinders) was determined in accordance with ASTM C-1202 [55] using a GERMANN PROOVEit apparatus system. For each formulation tested, three specimens were placed in cells which were containing 3% sodium chloride solution as well as 0.3 N sodium hydroxide solution. The samples were then subjected to a potential difference flow for a period of 6 h while the electric charge value (Coulombs) was monitored and recorded throughout the specific period. Resistivity measurements were taken on 9 points on the surface of the same cylindrical samples prior to them being tested for chloride resistance, in order to assess and validate the experimental results. The resistivity test was conducted using a 38 mm-probe PROCEQ Resipod surface resistivity meter by applying a current to the sample’s surface and measuring the potential difference between its outer probes.

For measuring the drying shrinkage of RAC, specimens of 300 × 75 × 75 mm dimensions were prepared and water cured for 28 days at 20 °C. The specimens were then placed in a conditioning environment (20 °C, 65% RH) throughout the remaining period of investigation and dimensional changes were obtained at weekly intervals using a length comparator apparatus for up to 20 weeks.

The sulfate resistance of the RAC was determined using a method proposed by Dhir et al. [56] which involves weekly monitoring of length changes of 28-day water cured 300 × 75 × 75 mm specimens, immersed in a 5% Na_2_SO_4_ solution at 20 °C. This exposure is approximately 5.6 times stronger than the most severe environment in EN 206-1 that suggests a SO_4_- concentration of 6 g/L. The solution was changed every 28 days, to maintain the concentrations through immersion period. Sulfate expansions were monitored throughout a period of 40 weeks.

Carbonation resistance of hardened concrete was measured in accordance to the EN 12390-12:2010 standard, where 300 × 75 × 75 mm samples were water cured at 20 °C for 28 days, followed by being conditioned in advance (20 °C, 65% RH) for a period of 14 days and placed in a carbonation chamber set to 4% CO_2_ atmosphere, 20 °C and 65% RH. Longitudinal slices of 50 mm were cut from the sample by using chisel and hammer at weekly intervals for the determination of carbonation depths. Phenolphthalein indicator solution was sprayed on the internal surface of the 50 mm slices while the remainder of the specimen was re-sealed and placed in the CO_2_ chamber. A total of 12 points were used to obtain the average carbonation depth, one hour after spraying.

### 2.3. Mixtures and Mixture Proportions

A total of 17 mixtures were developed based on the mix proportions shown in Table 3. The mixtures were prepared at w/c ratios 0.25 and 0.50 while the cement contents used were 400 kg/m^3^ and 864 kg/m^3^ in order to evaluate the effect of RCA in both normal and high strength concretes. The target consistency class was S3 in accordance with EN 206-1 [59]. For achieving this, a commercially available polycarboxylate-based superplasticizer was incorporated during the mixing and adjusted accordingly at 1–2% by weight of cement in the concrete mix. The mixtures were designed by incorporating 25%, 50% and 100% of RCA to NA by mass. The design for all mixtures was developed for achieving a similar all-in aggregate grading of the total aggregate content of the reference mixture. The code names of each mixture shown in Table 3 are provided below:

- “R” denoting recycled concrete aggregate
- “F” denoting the fine aggregates replacement (i.e., the second letter in nomenclature of each formulation)
- “C” denoting the coarse aggregates replacement
- “F” denoting the aggregate to be field, non-treated (i.e., the third letter in nomenclature of each formulation)
- “T” when aggregate is treated.
- “100”, “50” or “25” represent the replacement percentage of natural aggregates
- It should be noted that throughout the paper, the w/c ratio is suffixed at the end of each code name.

### 2.4. Casting and Curing

Mixes were produced in accordance with BS 1881-125:2013 [60] by using a Tecnotest AT 205 pan mixer (Tecnotest, Treviolo, Italy) of 150 L capacity. All investigated types of aggregates were maintained in the laboratory in air-dried condition and all necessary water content corrections were made based on their moisture content and absorption prior mixing. The samples were demoulded 24 h following casting and then water-cured at a constant temperature of 22 ± 2 °C until specified days of testing.

## 3. Results and Discussion

**Figure 2:**
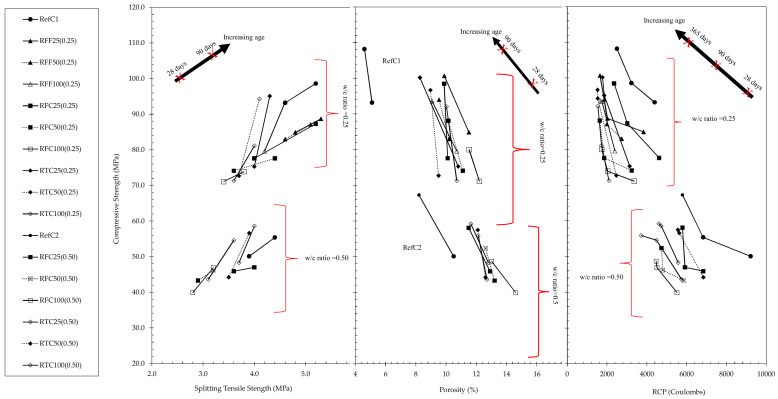
Relationship between compressive strength, porosity and chloride resistance of concrete mixtures.

**Figure 3:**
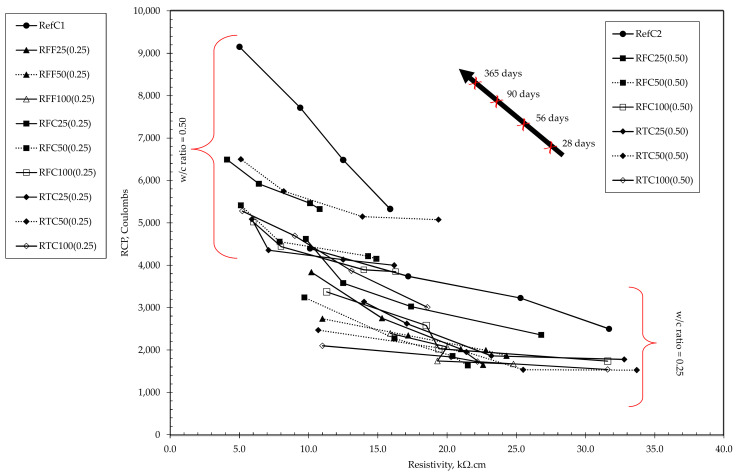
Relationship between chloride resistance and resistivity of concrete mixtures at 28, 56, 90 and 365 days.

The relationship between compressive and splitting tensile strength, porosity and chloride resistance of all investigated mixtures is shown in Figure 2. Compressive strength is an indicator, predominantly, of the evolution of the microstructural density of the cement paste, whereas split tensile strength is an indirect indicator of the bonding adherence between the aggregate and the cement matrix. The diagonal indication arrows appearing on top in Figure 2 show the increase in age of the samples, meaning that each data point plotted towards the indicated direction being the later age of testing. Based on the results, the incorporation of RCA reduced both compressive and splitting tensile strengths compared to the reference mixtures at both w/c ratios. In 90 days, however, the decrease in compressive strengths with respect to the reference strengths appeared to be smaller. In particular, it was observed that, regardless of the w/c ratio, the incorporation of treated coarse RCA at either 25%, 50%, or 100% by mass led to a sharper increase in strengths of the same mixtures from 28 days to 90 days, i.e., values were increased by almost 21–29% as observed by the slopes of the relevant curves; whereas untreated aggregates, when incorporated at any w/c ratio and at any percentage, led to a 5–10% compressive strength increase, i.e., a behaviour similar to that observed in reference mixtures. This may indicate that aggregate treatment affects less the early-age compressive strengths of high w/c ratio concretes, however it significantly enhanced the long-term mechanical properties at any w/c ratio. In all cases, compressive strengths were lowest at 100% replacements of either treated or untreated RCA (i.e., up to 24% strength reductions compared to the reference mixtures). Figure 3 also shows that the f_cu_/f_ct_ ratio was maintained relatively constant for all mixes from the 28th day to the 90th day, albeit at a lower value at higher w/c ratio mixtures (i.e., ranging between 11.8–15.2 in mixtures of 0.25 w/c ratio and 16.7–23.0 at w/c ratio of 0.5). An interesting point was nevertheless observed at higher w/c ratios. When treated coarse aggregates were incorporated at 25%, the 90-day strengths surpassed the compressive strengths of the reference by 5%. On the 50% replacement of the same, compressive strength was still 2% higher than those of the reference mixture, and this denotes that the treatment method enhanced the overall microstructural and density characteristics of the matrix, though such beneficial effect was restricted only up to a 50% replacement.

Treated recycled coarse aggregates, when incorporated in RAC of 0.25 w/c ratio, resulted in reduced compressive strengths at 90 days (up to 15% reduction from the reference) however, they caused more apparent reductions in splitting tensile strengths (up to 26.9% reduction compared to reference). The opposite appeared to occur in untreated fine/coarse RAC mixtures at low w/c ratios, where splitting tensile strengths at 90 days were maintained close to the reference, although compressive strengths were more drastically decreased, i.e., by 23.1%. Such behaviour would be probably related to the presence of unhydrated cement particles in the matrix as these low w/c ratio values incorporate inadequate water for hydration within the mixture. For the high w/c ratio concretes, where the differences in splitting tensile strengths was higher, this would be attributed to the presence of a double ITZ (i.e., the old and a new one). The results suggest that the effect of mechanical treatment of recycled aggregates on the bonding characteristics of RACs may be more beneficial to mixtures of higher w/c ratios where the cement content was lower, however, such effect had evidently benefited all treated RCA combinations at all w/c ratios when reached 90 days. In low w/c ratios at early ages, this effect was less apparent, which may indicate a possibility that the effect of geometrically altered aggregates in concrete could be somewhat hindered by the significantly high cement contents in the mixes. Nevertheless, the effect still appeared to have benefited even these mixtures at 90 days. When considering longer-term strengths (365 days) against porosity evolutions of the mixtures as shown in Figure 2, it still applied that the incorporation of treated RCA in the mixtures at both w/c ratios and at any replacement content caused an even sharper increase in strengths of RTC25/50/100(0.25, 0.50) i.e., on average a 25% increase, although reductions in porosities were not as correspondingly significant.

Significant outcomes were observed in the chloride resistance, resistivity and porosities investigation of the mixtures. All RAC mixtures appeared to have reduced the chloride penetration by up to 52% at low w/c ratios and 40% at high w/c ratios compared to the reference mixtures while having, interestingly, higher porosities than those of the reference mixtures. The addition of higher amounts of RCA in concrete mixes seemed to have increased the porosities of the mixtures, due to the presence of higher amounts of adhered mortar—which is a highly porous material. The reductions in both porosity and RCP values were well correlated with the reduction in w/c ratio as seen from Figure 2. In mixtures containing 100% replacement with either treated coarse RCA or untreated fine RCA, the reduction in the RCP at 28 and 90 days was significant, ranging between 40–50%, although as the samples matured (365 days), reductions were less (i.e., 33–38%). The same behaviour was observed in mixtures of high w/c ratio although improvements in chloride resistance were not as significant. When considering, however, the chloride resistance of REFC1 and REFC2, the exhibited values did not appear to fully correspond to their equivalent open porosity. Particularly, in the case of REFC1, the porosity values were significantly lower than the rest of the mixtures, reaching even 50% lower, while its corresponding RCP values did not seem to justify such pore network (i.e., 28-day and 90-day RCP values were the second-highest and still being comparably high at 365 days). The same behavior was observed for REFC2 where its 28-day RCP value appeared to be the highest of all by a notable difference while its porosity value was approximately 20% lower than the second lowest (i.e., RTC50 (0.5)). Such behavior may be associated with a number of probable mechanisms, either acting independently or in a synergistic manner—such as the formation of a primary and a secondary ITZ due to the presence of RCA, and/or the rapid formation of a shell during cement hydration due to high cement fineness, and the internal curing mechanism activated in mixtures containing recycled aggregates. It was also observed that the RCP results were cohering with the corresponding resistivity values as shown in Figure 3 on an inversely proportional relationship.

**Figure 4:**
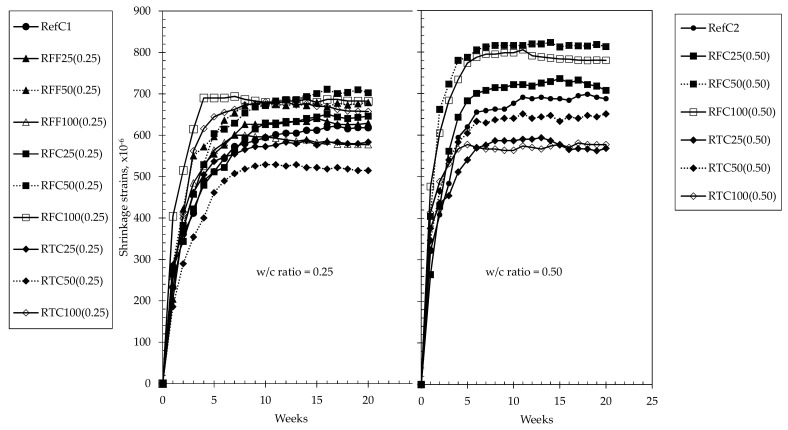
Drying shrinkage of concrete mixtures investigated up to 20 weeks.

**Figure 5:**
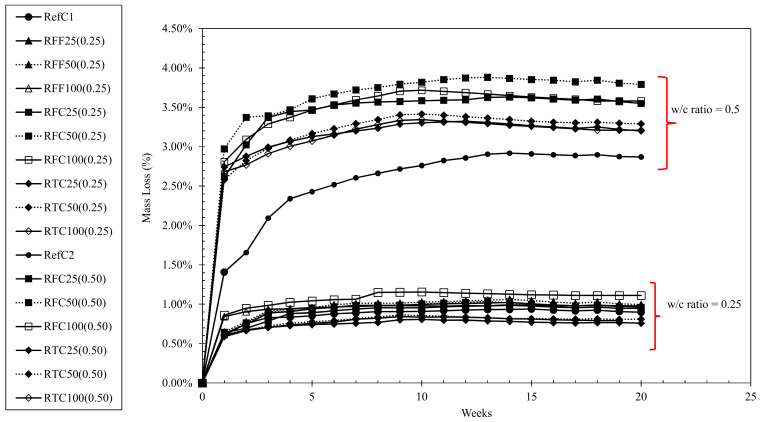
Mass loss measurements of concrete mixtures investigated for up to 20 weeks.

Drying shrinkage strains and mass loss of concretes examined for up to 20 weeks are shown in Figure 4 and Figure 5, respectively. The mixtures containing untreated (either fine or coarse) RCA exhibited higher strains than those of the reference at any w/c ratio, and, at higher replacement percentages of RCA, a 13–18% increase in shrinkage strains occurred compared to the reference mixtures. While highest values were recorded at 50% and 100% replacement percentage of untreated coarse RCA at both w/c ratios, it was observed that concretes containing treated coarse RCA—and particularly RTC50(0.25), RTC25(0.50) and RTC100(0.50) exhibited reduced shrinkage strains by 14–18% at both w/c ratios. Similar behaviour was observed in the mass loss results although this was apparent only in the case of w/c ratio of 0.25, where RTC100 (0.25) and RTC50(0.25) maintained an approximately 10% lower mass loss than that of RefC1.

**Figure 6:**
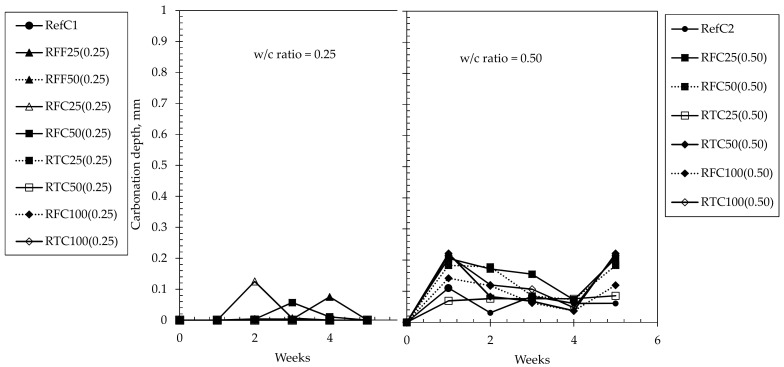
Carbonation depths of concrete mixtures examined up to 5 weeks.

Another observation made was that in most mixtures, shrinkage became dormant approximately at the 8th week of testing, although mass losses were still continuing to occur up to even the 10th week in some mixtures. Based on Figure 4, it can be therefore suggested that a 3-h mechanical treatment provided a shrinkage-controlling effect in RAC, and particularly to those of low w/c ratios. Based on Table 1, treated RCA are of better quality and they provided higher restraint to shrinkage. Another interesting point observed was the similar strain magnitudes in both w/c ratios, while mass losses were affected significantly by w/c ratio. For the same w/c ratio, drying shrinkage increases as the amount of cement increases. For a constant amount of cement, drying shrinkage increases as the w/c ratio increases. In this case, w/c decreases (0.50 to 0.25) but at the same time the amount of cement increases (400 kg to 846 kg), therefore, comparable results should not be unexpected. Carbonation resistance of concretes at both w/c ratios of 0.25 and 0.50 when exposed throughout a 5-week period is shown in Figure 6 for both w/c ratios. It was observed that all concretes of w/c ratio of 0.25 exhibited depths almost equal to zero throughout the full testing period, whereas concretes at w/c ratio of 0.50 yielded slightly higher, and almost constant values throughout the testing period. The results suggest that the carbonation resistance of RACs is comparable to that of the reference mixtures, while the amount, size and type of RCA incorporated in the mixtures did not appear to be parameters that affect such property. Fundamentally, the mechanism of carbonation relies on the reaction between CO_2_ and the available Ca(OH)_2_ in concrete in the presence of humidity, to yield CaCO_3_ and alter the molar volume of the matrix while further minor phases may additionally incur such as calcium carboaluminates produced from the AFm phases. When considering the investigated mixtures, the amounts of alkalis present are significantly high as of the large volumes of CEM I 52.5N and these are backed by the high pH values in the pore solution. This fact appears to be the major and most predominant factor controlling the carbonation resistance of concrete while the w/c ratio, based on the figures, seemed to be evidently the second parameter that defines the magnitude of the carbonation depths. Even though the carbonation depths of concretes at w/c 0.5 were higher than their corresponding RefC1 concrete and higher those of w/c ratio of 0.25, still however, these values may be considered insignificant (less than 0.25 mm) when comparing to previous research, and the carbonation resistance of RACs is considered comparable to that of their REF equivalents. An interesting fact noted was that the incorporation of treated aggregates at 25% by mass within w/c 0.5 mixtures improved the carbonation resistance making it comparable to that of RefC2 concrete, although the aforementioned differences were not significant compared than the rest of the mixtures with w/c ratio of 0.5. Furthermore, the results showed that the w/c ratio along with the cement content were more predominant factors defining the carbonation resistance than incorporating and/or treating RCA within the concrete. Any formations of secondary ITZs or any weaker bonding between RCA and mortar did not occur to a degree that would hinder the beneficial effect that the present alkali hydroxyls provided within the concrete.

**Figure 7:**
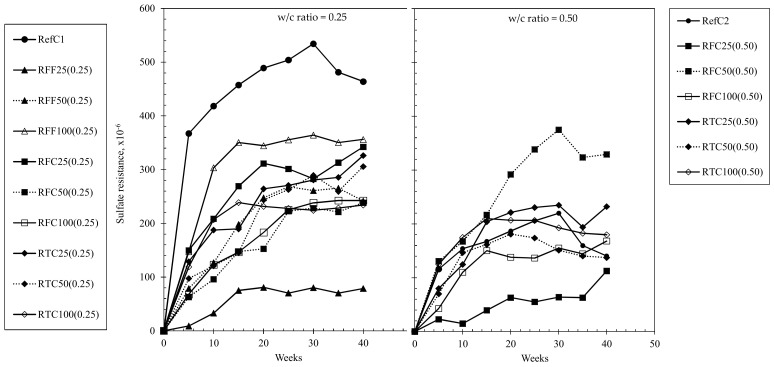
Sulfate resistance of concrete mixtures examined up to 40 weeks in 5% NaSO_4_ solution.

The sulfate resistance of concretes investigated up to 40 weeks of exposure at both w/c ratios is shown in Figure 7. It can be clearly seen that all samples, regardless of the w/c ratio, appeared to have undergone shrinkage deformations during the first 15 weeks, and certain mixtures including RefC1, continued to exhibit shrinkage beyond that timeframe. Mixtures of 0.5 w/c ratio exhibited even up to 58% lower shrinkage strains than those of 0.25 w/c ratio and there appeared to be no clear correlation between the replacement percentage of RCA, or the treatment effect of RCA against the deformations. Notwithstanding the above, the results suggest that the impact of sulfate attack was slightly significant, however, it would still most probably cause no major concerns or risks associated with expansive ettringite formation in either the high or the low w/c ratio RAC with treated or untreated RCA.

Fundamentally, the mechanism of sulfate attack is based on the reaction between sulfate ions and the available Ca (OH)_2_, C3A and AFm phases in concrete to form expansive ettringite, which in turn increases the molar volume of the matrix causing internal stresses, cracking and mass loss. The permeability of concrete is a significant factor affecting the penetration of sodium sulfate solution in the matrix [61]. Because the incorporation of either treated or untreated RCA essentially affects the interactions in the ITZ as well as the bonding, deformation and restraint characteristics of concrete and while RCA do not cause any significant alterations in the phase formations of the cement paste, then it follows that little effects would occur related to the expansion due to sulfate attack mechanism although it may have an impact on the compressive strength and mass loss of RACs [61,62].

## 4. Conclusions

A comprehensive long-term mechanical properties and durability assessment was conducted on RAC mixtures incorporating untreated, as well as treated RCA that had undergone mechanical treatment. Following the treatment process, the aggregates were utilized in concrete combinations at w/c ratios 0.50 and 0.25, with strategic replacement percentages of fine, coarse treated and untreated aggregates at 25%, 50% and 100% by mass. The following conclusions were drawn:

- The incorporation of 3-h mechanically treated RCA at 25%, 50% and 100% replacement levels offered a sharper increase in long-term compressive strengths than any other combination or reference mixture investigated in the study. The uprises in strength from 28 to 90 to 365 days reached a range of approximately 25%. Such differences were more drastic than those in porosities of the same combinations.
- An optimal mechanical treatment process appeared to offer a ‘shrinkage-controlling’ effect in treated RACs, where shrinkage strains of up to 20 weeks were reduced by almost 15% compared to reference concrete, and this was also reflected in the mass loss measurements.
- Optimal treatment of RCA in concrete initially appeared to have, to a lesser extent, affected the early-age compressive strengths and porosities of the investigated mixtures; then, it appeared to be benefitting only the strengths of high w/c ratio mixtures; and finally, it enhanced the long-term mechanical properties of both w/c ratios.
- The carbonation resistance and sulfate resistance of RAC appeared to be comparable to those of reference mixtures. As these two aspects are predominantly defined by the hydration products available within the cement paste and to a lesser extent by the aggregate quality, it follows that the incorporation of either treated or untreated RCA in concrete did not appear to expose RACs to any durability concerns.
- The reductions in both porosity and RCP values were well correlated with the reduction in w/c ratio. In mixtures containing 100% replacement with either treated coarse RCA or untreated fine RCA, the reduction in the RCP at 28 and 90 days was significant, ranging between 40–50%, although as the samples matured (365 days), reductions were less (i.e., 33–38%). The same behaviour was observed in mixtures of high w/c ratio although improvements in chloride resistance were not as significant.

## Figures and Tables

**Table 1:** Characterization of aggregates used in the experimental program.

Properties	Relevant Standard	0/2 mm NA	4/10 mm NA ^1^	4/10 mm RTC ^2^	4/10 mm RFC ^3^	0/4 mm NA	0/4 mm RFF ^4^	8/20 mm NA	8/20 mm RFC	8/20 mm RTC
Los Angeles Coefficient (LA) (%)	ASTM C131 [47]	-	29	15	32	-	-	29 *	32 *	15 *
Particle Density (Kg/m^3^)	EN 1097-6 [48]	2530	2473	2430	2517	2267	2299	2500	2430	2400
Particle Density, SSD (Kg/m^3^)	EN 1097-6 [48]	2580	2567	2539	2681	2378	2413	2600	2530	2490
Water Absorption (%) (WA) (%)	EN 1097-6 [48]	1.80	3.79	4.48	6.52	4.89	4.95	4.10	4.40	4.00
Soundness (%)	ASTM C88 [49]	-	30	14	41	-	-	30 *	41 *	14 *
Flakiness Index	EN 933-3 [50]	-	16	4	5	-	-	7	5	6
Shape Index	EN 933-4 [51]	-	9	5	7	-	-	9	16	15

^1^ NA = Natural Aggregates; ^2^ RTC = Recycled Treated Coarse Aggregate; ^3^ RFC = Recycled Field Coarse Aggregate (untreated); ^4^ RFF = Recycled Field Fine Aggregate (untreated).; * Values from Dimitriou et al. [30].

**Table 2:** Experiments, age of testing, specimen size and standards used in the experimental program.

Test			Standard	Age of Testing (Days)	Specimens
Hardened Concrete properties	Mechanical	Compressive Strength	EN 12390-3 [53]	28, 90, 365	100 mm cubes
		Tensile Splitting Strength	EN 12390-6 [54]	28, 90	100Φ200 mm cylinders
	Durability	Carbonation Resistance	EN 12390-12 [57]	Weekly up to 5 weeks	300 × 75 × 75 mm prism
		Open Porosity	[7]	28, 90, 365	100 mm cubes
		Drying Shrinkage	EN 1367 [58]	Weekly up to 20 weeks	300 × 75 × 75 mm prism
		Sulfate Resistance	Reference [56]	Weekly up to 40 weeks	300 × 75 × 75 mm prism
		Chloride Ion Resistivity	ASTM C-1202 [55]	28, 56, 90, 365	100Φ200 mm cylinders

**Table 3:** Mix proportions, nomenclature and replacement percentages of RCA in concrete mixes.

		Water	Portland Cement	Sand 0/2 Mm	Sand 0/4 Mm	Aggregate 4/10 Mm	Aggregate 8/20 Mm	Recycled Sand 0/4 Mm	Recycled Aggregate 4/10 Mm	Recycled Aggregate 4/10 Mm	Recycled Aggregate 8/20 Mm	Recycled Aggregate 8/20 Mm
	Code	-	PC	-	-	NA 4/10 mm	NA 8/20 mm	RFF 0/4 mm	RFC 4/10 mm	RTC 4/10 mm	RFC 8/20 mm	RTC 8/20 mm
w/c Ratio	Description	-	CEM I 52.5N to EN197-1	Natural Fine Sand	Natural Diabase Sand	Natural Crushed Aggregate	Natural Crushed Aggregate	Field Sand (Untreated)	Field Aggregate (Untreated)	Mechanically Treated Aggregate	Field Aggregate (Untreated)	Mechanically Treated Aggregate
		Constituent Contents in Mix Design (kg/m^3^)										
0.25	REFC1	216	864	184	335	730	-	-	-	-	-	-
	RFF25	216	864	183	251	730	-	84	-	-	-	-
	RFF50	216	864	184	167	730	-	167	-	-	-	-
	RFF100	216	864	183	-	730	-	334	-	-	-	-
	RFC25	216	864	183	334	548	-	-	183	-	-	-
	RFC50	216	864	184	334	365	-	-	365	-	-	-
	RFC100	216	864	184	335	-	-	-	730	-	-	-
	RTC25	216	864	184	335	547	-	-	-	182	-	-
	RTC50	216	864	184	335	365	-	-	-	365	-	-
	RTC100	216	864	184	334	-	-	-	-	730	-	-
0.50	REFC2	200	400	406	266	386	629	-	-	-	-	-
	RFC25	200	400	407	266	289	471	-	96	-	157	-
	RFC50	200	400	407	266	192	314	-	192	-	314	-
	RFC100	200	400	407	266	-	-	-	385	-	628	-
	RTC25	200	400	406	266	289	471	-	-	96	-	157
	RTC50	200	400	406	266	193	314	-	-	193	-	314
	RTC100	200	400	406	266	-	-	-	-	385	-	629

## Data Availability

Not applicable.
